# Rosiglitazone Increases Cerebral *Klotho* Expression to Reverse Baroreflex in Type 1-Like Diabetic Rats

**DOI:** 10.1155/2014/309151

**Published:** 2014-02-13

**Authors:** Li-Jen Chen, Meng-Fu Cheng, Po-Ming Ku, Jia-Wei Lin

**Affiliations:** ^1^Institute of Basic Medical Science, College of Medicine, National Cheng Kung University, Tainan 70101, Taiwan; ^2^Division of Nephrology, Department of Internal Medicine, College of Medicine, National Cheng Kung University, Tainan 70101, Taiwan; ^3^Department of Cardiology, Chi-Mei Medical Center, Yong Kang, Tainan 71004, Taiwan; ^4^Department of Neurosurgery, Taipei Medical University-Shuang Ho Hospital, Taipei 23561, Taiwan

## Abstract

Reduced baroreflex sensitivity (BRS) is widely observed in diabetic human and animals. Rosiglitazone is one of the clinically used thiazolidinediones (TZD) known as PPAR**γ** agonist. Additionally, the klotho protein produced from choroid plexus in the central nervous system is regulated by PPAR**γ**. In an attempt to develop the new therapeutic strategy, we treated streptozotocin-induced diabetic rats (STZ) with rosiglitazone (STZ + TZD) orally at 10 mg/kg for 7 days. Also, STZ rats were subjected to intracerebroventricular (ICV) infusion of recombinant klotho at a dose of 3 **μ**g/2.5 **μ**L via syringe pump (8 **μ**g/hr) daily for 7 days. The BRS and heart rate variability were then estimated under challenge with a depressor dose of sodium nitroprusside (50 **μ**g/kg) or a pressor dose of phenylephrine (8 **μ**g/kg) through an intravenous injection. Lower expression of klotho in medulla oblongata of diabetic rats was identified. Cerebral infusion of recombinant klotho or oral administration of rosiglitazone reversed BRS in diabetic rats. In conclusion, recovery of the decreased klotho in brain induced by rosiglitazone may restore the impaired BRS in diabetic rats. Thus, rosiglitazone is useful to reverse the reduced BRS through increasing cerebral klotho in diabetic disorders.

## 1. Introduction

Cardiovascular complications influence the prognosis of diabetic disorders and became the main reason for the high mortality of diabetic patients [1, 2]. A number of studies have reported that diabetes mellitus (DM) leads to impairment of the baroreflex control of heart rate (HR) in both diabetic patients and animals. Impairment of the baroreflex sensitivity (BRS) underlying the diabetic state is closely related to life-threatening arrhythmias, heart failure, and sudden death [3, 4].

To minimize the short-term fluctuations of blood pressure and maintain a homeostatic state, a negative feedback system called the baroreflex buffers these short-term changes such as an increased blood pressure which results in slower heart rate and vice versa [5]. Reduced BRS has been characterized in diabetic human [6, 7] and in diabetic rats induced by streptozotocin (STZ) as type 1-like diabetic model [8, 9].

Many factors related to the baroreflex have been mentioned in the central nervous system. The higher baroreflex gain induced by angiotensin has been characterized in rats after an intracerebroventricular (ICV) injection. The development of hypertension in DOCA-salt rats and/or the disorders of chronic heart failure (CHF) were both reduced under higher baroreflex [10, 11]. ICV infusion of leptin ameliorated the variability of heart rate (HR) and the baroreflex sensitivity in STZ-induced diabetic rats [12]. Thus, regulation of baroreflex sensitivity from central nervous system seems important.

The *klotho* gene has been suggested in 1997 using *klotho* mutant mice (*kl/kl* mice) with multiple disorders similar to human premature-aging syndrome [13]. Also, a single nucleotide polymorphism in human *klotho* gene has been mentioned to be associated with the development of hypertension in both Chinese Han [14, 15] and Caucasoid [16–19] subjects. In *kl/kl* mice, alterations occurred mainly in a cell-non-autonomous fashion, while *klotho *gene expression is predominantly shown in kidney, parathyroid gland, and choroid plexus, although the gene is not expressed in other organs that can be markedly influenced [13]. The choroid plexus may produce cerebrospinal fluid (CSF) for neurons and the secreted klotho protein was observed in CSF [20]. Thus, the klotho in brain can be considered to play a role in the regulation of cardiovascular homeostasis.

Klotho has been mentioned as the target of PPAR*γ* [21]. In clinics, agent of TZDs has been used to treat type 2 diabetic patients through correction of both hyperlipidemia and hyperglycemia [22, 23]. Also, TZDs ameliorate the renal injury in STZ-diabetic rats through the anti-inflammatory action [24] or increased PPAR*γ* expression [25]. The renal protection in STZ-diabetic rats by TZDs produced without altering the blood glucose level [26]. Since klotho was the target of PPAR*γ* [21], the higher klotho expression by PPAR*γ* may result in the improvement of dysfunctions in STZ-diabetic rats. The widely used TZDs, including pioglitazone, rosiglitazone, and troglitazone, were demonstrated to increase the expression of klotho, while rosiglitazone was most effective [21]. Thus, we employed rosiglitazone as the representative of TZDs in this study.

The present study is designed to know whether rosiglitazone (TZD) ameliorates the impaired baroreflex sensitivity (BRS) in STZ-induced diabetic rats. We also determined the mediation of klotho in this action because rosiglitazone is known to increase klotho [27]. Then, the role of klotho in BRS could be elucidated in diabetic state.

## 2. Material and Methods

### 2.1. Animals

The male Wistar rats, weighing from 200 to 250 g, were obtained from the Animal Center of National Cheng Kung University Medical College. All rats were housed individually in plastic cages under standard laboratory conditions. They were maintained under a 12 h light/dark cycle and had free access to food and water. All experiments were performed under anesthesia with 2% isoflurane, and all efforts were made to minimize the animals' suffering. The animal experiments were approved and conducted in accordance with local institutional guidelines for the care and use of laboratory animals in Chi-Mei Medical Center (number. 100052307) and the experiments conformed to the Guide for the Care and Use of Laboratory Animals as well as the guidelines of the Animal Welfare Act.

### 2.2. Streptozotocin (STZ) Induced Type 1-Like Diabetic Rats

Diabetic model was induced by an intravenous (i.v.) injection of STZ (Sigma-Aldrich Inc., USA) at 65 mg/kg into the fasting Wistar rats as described previously [28]. The animals were considered to be diabetic if they showed a plasma glucose concentration over 350 mg/dL in addition to the diabetic syndromes.

The plasma glucose levels were measured in blood samples collected from the femoral veins of anesthetized rats. Body weight was also monitored during the experiment. At the end of treatment, animals were sacrificed, and the tissues were dissected, washed with saline, and weighed. For further analysis, samples were frozen in liquid nitrogen for storage at −80°C.

Blood samples from rats were centrifuged at 12,000 g for 3 minutes. Samples were then analyzed using the glucose kit reagents (AppliedBio assay kits; Hercules, CA, USA). The level of plasma glucose was then estimated using an autoanalyzer (Quik-Lab, USA) and measured in duplicate.

### 2.3. Drug Administration

The age-matched rats were divided into three groups (*n* = 8): vehicle-treated normal rats (Wistar); vehicle-treated STZ rats (STZ); and TZD-treated STZ rats (STZ + TZD) through oral intake of 10 mg/kg rosiglitazone (Avandia) daily for seven days as described previously [29, 30].

### 2.4. Western Blotting Analysis

Protein was extracted from tissue homogenates using ice-cold radioimmuno-precipitation assay (RIPA) buffer supplemented with phosphatase and protease inhibitors (50 mmol/L sodium vanadate, 0.5 mM phenylmethylsulphonyl fluoride, 2 mg/mL aprotinin, and 0.5 mg/mL leupeptin). The protein concentrations were determined using a Bio-Rad protein assay (Bio-Rad Laboratories, Inc., Hercules, CA, USA). Total proteins (30 *μ*g) were then separated using SDS/polyacrylamide gel electrophoresis (10% acrylamide gel) through a Bio-Rad Mini-Protean II system. The protein was transferred to expanded polyvinylidene difluoride membranes (Pierce, Rockford, IL, USA) with a Bio-Rad Trans-Blot system. The membrane was blocked with 5% nonfat milk in phosphate-buffered saline containing 0.1% Tween 20 (PBS-T) and incubated for two hours. The membrane was then washed in PBS-T and hybridized with primary antibodies, specific antibodies for klotho, which were diluted to a suitable concentration (1 : 1000) in PBS-T for 16 hours. Incubation with secondary antibodies and detection of the antigen-antibody complex were performed using an ECL kit (Amersham Biosciences, UK). The immunoblot densities at 130 KD were quantified using a laser densitometer. Expression of *β*-actin was used as the internal standard.

### 2.5. Intracerebroventricular (ICV) Injection

Following to the previous method [12], the well-anesthetized rats were immobilized in a stereotaxic frame to prepare for ICV injection. Then, the age-matched rats were divided into four groups (*n* = 8): normal rats (Wistar); STZ rats (STZ); rat IgG (IgG)-treated STZ rats (STZ + IgG); and recombinant klotho (rKl)-treated STZ rats (STZ + rKl). Rat rKl or rat IgG (Abcam, Cambridge, MA, USA) was dissolved in artificial cerebrospinal fluid (ACSF) at a dose of 3 *μ*g/2.5 *μ*L for ICV infusion using syringe pump (Harvard Apparatus, Holliston, MA, USA) (8 *μ*g/hr) for seven days according to the previous reports [12, 31, 32].

### 2.6. Arterial Pressure and Heart Rate Recording

The rats were anesthetized with 2% isoflurane, and a catheter was inserted into the femoral artery for recording of blood pressure and heart rate. The catheters were made of 4 cm segments of PE-10 polyethylene (Clay Adams, USA) that was heat-bound to a 13 cm segment of PE-50 (Clay Adams, USA). After surgery, the animals were allowed 20 min to adapt the experimental conditions, such as sound and illumination. Another 15 min period was allowed before beginning of experiment. The pressure catheter was connected to an external computer (IX-214; iWorx Systems, Inc., Dover, NH, USA) to acquire all signals. The mean arterial pressure (MAP) and heart rate (HR) were derived from Labscribe2 (iWorx Systems, Inc., Dover, NH, USA) [33, 34].

### 2.7. Baroreflex Challenge and Evaluation

According to previous methods [33, 34], the baroreflex response was challenged using a pressor dose of 0.1 mL phenylephrine (PE; 8 *μ*g/kg IV) or a depressor dose of 0.1 mL sodium nitroprusside (SNP; 50 *μ*g/kg IV). The baroreflex sensitivity (BRS) was then calculated as the derivative of the HR in the function of the MAP variation (ΔHR/ΔMAP). The bradycardic and tachycardic peaks were also analyzed to determine the HR range and the difference between two peaks, as described previously [33, 34].

### 2.8. Statistical Analysis

All data are expressed as the mean ± standard error (SE) of each group. Using the Microsoft excel, statistical analysis was performed by the one-way ANOVA and the significance was obtained from the level at 2*α* = 0.05.

## 3. Results

### 3.1. Klotho Expression in the Medulla Oblongata of STZ-Diabetic Rats

**Figure 1:**
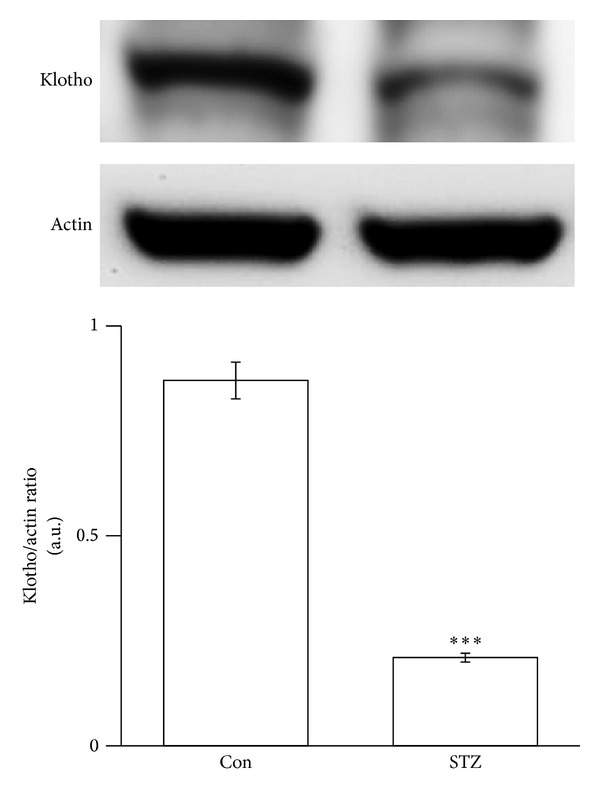
Klotho protein expressions in the medulla oblongata of STZ-diabetic rats. Expression of klotho protein (130 kDa) in Wistar rats (Wistar) and STZ-diabetic rats (STZ) was identified using Western blotting analysis. Samples were prepared from the medulla oblongata. The corresponding *β*-actin (Actin) protein level was used as an internal control. The quantification of protein levels was expressed as klotho over *β*-actin. The quantification is indicated as the means with the SE (*n* = 8 per group) in each column shown in the lower panel. ***P < 0.001 compared to Wistar.

Expressions of the klotho protein in the medulla oblongata between Wistar rats and STZ-diabetic rats were compared using Western blotting analysis. The expression of klotho protein in the medulla oblongata was significantly lower in STZ-diabetic rats than normal Wistar rats (Figure 1).

### 3.2. ICV Injection of Recombinant klotho Restored the Baroreflex Responses in STZ-Diabetic Rats

**Figure 2:**
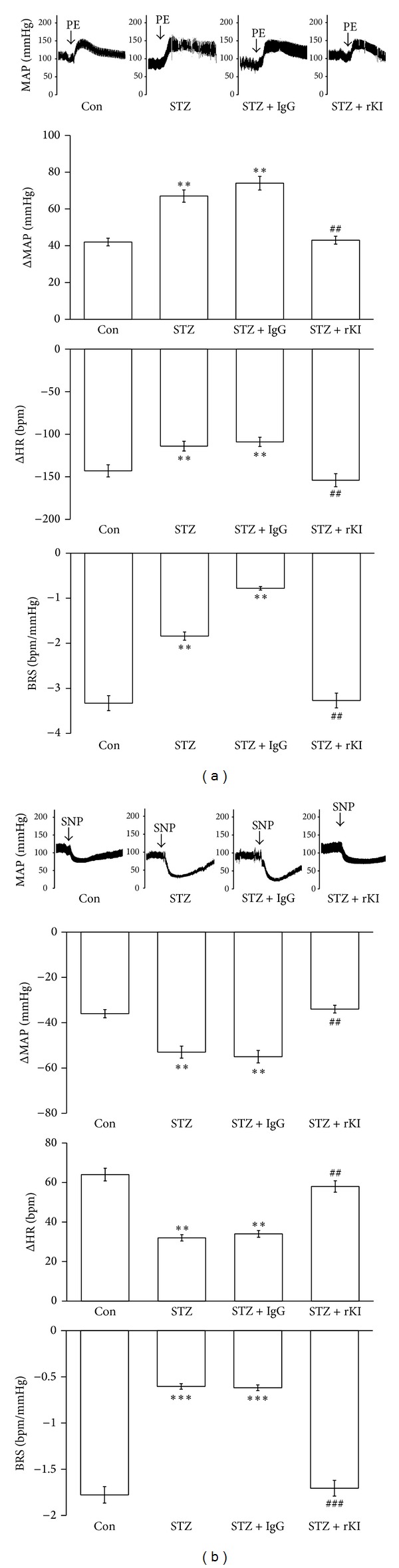
Effect of recombinant klotho (rKl) on the sensitivity of the baroreflex in STZ-diabetic rats. The effect of rKl or rat IgG (IgG) on the mean arterial pressure (MAP, mmHg), heart rate (HR, bpm), and baroreflex sensitivity (BRS, bpm/mmHg) in response to phenylephrine (PE, 8 *μ*g/kg, IV) (a) or (SNP, 50 *μ*g/kg, IV) (b) in each group; Con means control, STZ shows STZ-diabetic rats, STZ + IgG is IgG-treated group and STZ + rKl indicates the recombinant klotho-treated STZ group. The quantification is indicated as the means with the SE (*n* = 8 per group) in each column shown in the lower panel. **P < 0.01 and ***P < 0.001 compared to Wistar.

As shown in Table 1, the basal MAP and HR were markedly different between STZ-diabetic rats and Wistar rats. Additionally, after challenging the baroreflex, there is no marked difference on the values of bradycardic peak between Wistar and STZ group. However, the tachycardic peak was significantly reduced in STZ-diabetic rats, the values of HR range were markedly lower in STZ-diabetic rats than in normal Wistar rats. These changes in STZ group were restored by the ICV infusion of rKl, but they were not modified by IgG infusion, without altering the blood glucose level. The baroreflex gain resulting from challenge with PE or SNP was significantly reduced in the STZ group. This decrease in the baroreflex gain was also restored by the ICV infusion of rKl (Figures 2(a) and 2(b)).

### 3.3. Effect of TZD on the Expression of klotho in Medulla Oblongata of STZ Rats

**Figure 3:**
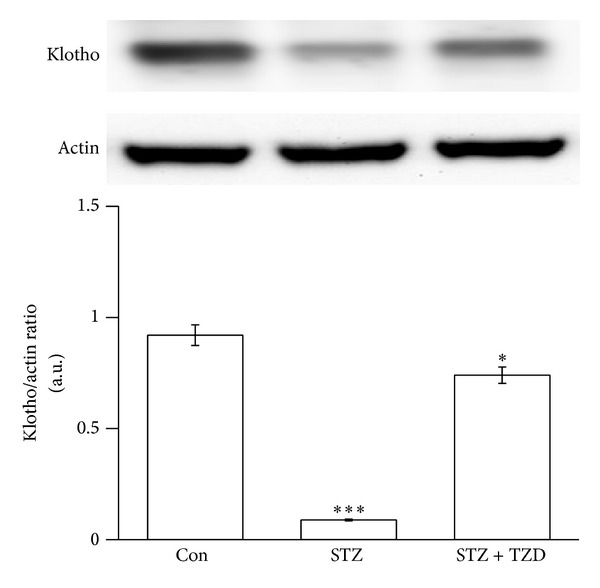
Effect of TZD on the expression of klotho in the medulla oblongata of STZ-diabetic rats. The upper shows the klotho protein level (Klotho) or the corresponding *β*-actin (Actin) level as internal control in the medulla oblongata isolated from Wistar rats (Con) and STZ-diabetic rats receiving TZD (STZ + TZD) or not (STZ). The treatments are described in materials and methods. Quantification of protein levels using klotho over *β*-actin to show the means with SE (*n* = 8 per group) in each column are indicated in the lower panel. **P < 0.01 and ***P < 0.001 compared to control (Con).

Changes of klotho expression in the medulla oblongata from STZ-diabetic rats were also identified using Western blots. After oral administration of rosiglitazone (TZD) for 7 days, the decreased klotho expression was also significantly reversed in the medulla oblongata of STZ-diabetic rats (Figure 3).

### 3.4. The Baroreflex Response is Restored in STZ Rats after Oral Administration of TZD

**Figure 4:**
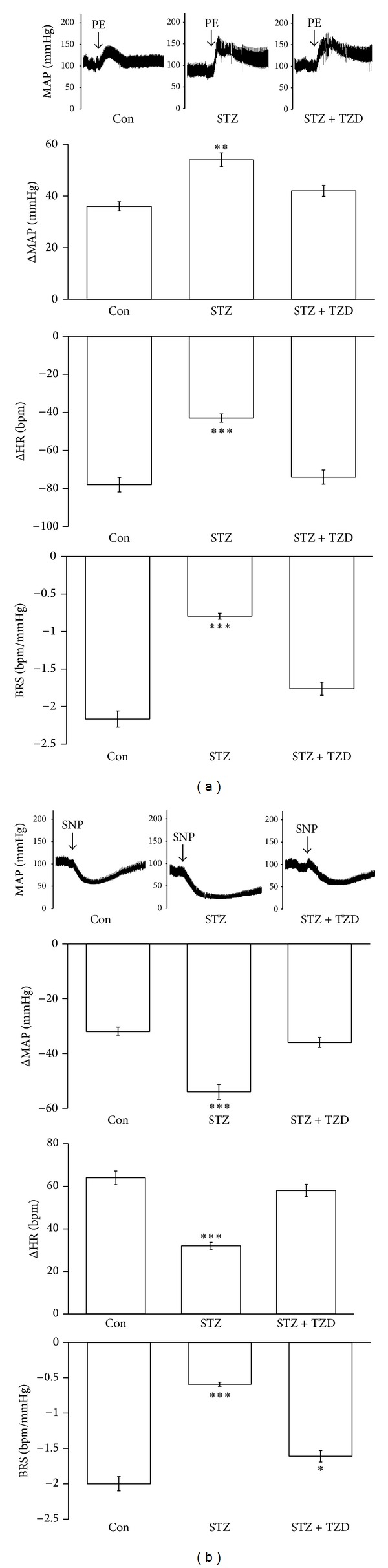
Effect of TZD on the sensitivity of the baroreflex in STZ-diabetic rats. The effect on the mean arterial pressure (MAP, mmHg), heart rate (HR, bpm), and baroreflex sensitivity (BRS, bpm/mmHg) in response to phenylephrine (PE, 8 *μ*g/kg, IV) (a) or (SNP, 50 *μ*g/kg, IV) (b) in each group including Wistar rats (Con) and STZ-diabetic rats receiving TZD (STZ + TZD) or not (STZ). The quantification is indicated as the means with the SE (*n* = 8 per group) in each column shown in the lower panel. **P < 0.01 and ***P < 0.001 compared to control.

As shown in Table 2, the basal MAP and HR were significantly normalized in the STZ-diabetic rats treated with rosiglitazone (TZD). Additionally, there is no marked difference on the values of the bradycardic peak between each group. However, the tachycardic peak was significantly restored in the STZ + TZD group as compared to STZ group and showing a significant difference in the HR range. The baroreflex gain resulting from PE or SNP challenge was both restored in the STZ + TZD group (Figures 4(a) and 4(b)). PE-induced increase of MAP was lower in STZ + TZD group than that in STZ group. Then, the bradycardic reflex responses to PE were markedly lower in the STZ + TZD group than that in the STZ group (Figure 4(a)). Intravenous injection of SNP produced a vasodepressor response; the value of MAP was still higher in STZ + TZD group than in STZ group. Moreover, the tachycardic reflex in response to SNP challenge was restored in STZ + TZD group (Figure 4(b)). Thus, the baroreflex gain was significantly restored in STZ-diabetic rats treated with TZD without altering the blood glucose level.

## 4. Discussion

In the present study, we demonstrate, for the first time, that the expression of klotho protein was significantly lower in the medulla oblongata of STZ-diabetic rats than normal Wistar rats. The baroreflex gain in response to challenge with PE or SNP was also reduced in STZ-diabetic rats compared to normal rats. We infused recombinant klotho into the brain of this type 1-like diabetic animal to restore the baroreflex responses without correcting blood glucose. Additionally, the expression klotho was significantly reversed in diabetic rats receiving rosiglitazone (TZD). The baroreflex responses triggered by PE or SNP were also increased in STZ-diabetic rats treated with rosiglitazone without changing blood glucose level. Thus, rosiglitazone has an ability to restore the reduced baroreflex responses through increase of cerebral klotho in diabetic rats.

Mutation of klotho may result in many aging-related disorders in animals; the expression of *klotho* gene is only identified in some tissues in mice, rats, and humans [35]. The klotho is documented to predominantly express in kidney and choroid plexus of the brain, although a slight expression of klotho has also been observed in the pituitary gland, placenta, skeletal muscle, colon, urinary bladder, pancreas, testis, ovary, and inner ear [13, 36]. CSF from choroid plexus is known to serve as the extracellular fluid for neurons [37]. Thus, klotho protein is suggested as a humoral factor [38] and it is detectable in CSF [20]. In the present study, we observed the reduced expression of klotho in the medulla oblongata of diabetic rats and this view has not been mentioned before.

Single nucleotide polymorphisms of the human *klotho* gene are associated with the development of cardiovascular diseases in both Chinese Han [14, 15] and Caucasoid [16–19] subjects. Baroreflex dysfunction observed in diabetic subjects has important clinical implications, because the baroreflex included an important system that acts against wide oscillations in arterial pressure (AP). Additionally, clinical trials have shown an association between baroreflex dysfunctions [39, 40]. Studies using experimental animals have been conducted to investigate the mechanisms of cardiovascular reflex dysfunction in diabetes [41]. It has been demonstrated that, in STZ-induced experimental diabetes, baroreflex control of circulation was impaired. In this study, we provide the first demonstration of a marked decrease of klotho in the medulla oblongata of STZ-diabetic rats. Also, we challenged the baroreflex response using a pressor dose of 0.1 mL phenylephrine (PE; 8 *μ*g/kg IV) or a depressor dose of 0.1 mL SNP (50 *μ*g/kg) according to previous reports [33, 34]. The baroreflex sensitivity (BRS) was calculated as the derivative of the HR in function of the MAP variation (ΔHR/ΔMAP). The tachycardic and the bradycardic peaks were also analyzed. The basal MAP and HR were significantly lower in STZ group than in the normal group. The pressor responses to PE were more marked in the STZ group than in the normal group, whereas the bradycardic reflex was reduced in the STZ group. The baroreflex gain was also attenuated in the STZ group. Similar changes were noted in the SNP-challenged STZ group. These results are consistent with a previous report [27]. However, there was no difference on bradycardiac peak between normal and STZ groups. The baseline heart rate values in STZ rats were alternatively lower than that in normal rats. It is may be the reason why there is no differences in bradycardiac peak between normal and STZ groups. However, the heart rate range still decreased in STZ group. Additionally, the decreased baroreflex responses were restored by the recombinant klotho infused into the brains of STZ group by ICV injection. Thus, increase of cerebral klotho appears to be useful in the recovery of the lowered baroreflex sensitivity.

Actually, STZ-diabetic rats treated with rosiglitazone result in an increase of klotho expression in the medulla oblongata (Figure 3). Then, we evaluated the effect of rosiglitazone on baroreflex responses in STZ-diabetic rats. The basal MAP and HR in diabetic rats were both increased by rosiglitazone near to the values in normal rats. Additionally, the pressor responses to PE were reduced to normal rats in the STZ + TZD group; the bradycardic reflex and baroreflex gain were both restored in the STZ + TZD group. Similar changes were also noted in the SNP-challenged baroreflex gain in STZ-diabetic rats. Thus, treatment of rosiglitazone or TZD seems beneficial in the recovery of baroreflex sensitivity in STZ-diabetic rats without changing blood glucose. Taken together, we demonstrated that the higher the expression of klotho by rosiglitazone the more restored the baroreflex in STZ-diabetic rats. However, the molecular mechanisms underlying the regulation of the baroreflex by klotho remain unclear and it needs more investigations in the future. Rosiglitazone has been demonstrated to be able to cross the blood brain barrier and that it is not exported out of the brain [42]. Some studies also demonstrated the neuroprotective merits of TZDs in animal models including focal ischemia, Parkinson's disease, and ALS [43–46]. The neuroprotective effects are introduced to be associated with PPAR*γ* mediated suppression of the inflammatory pathway [47] or by increasing antioxidant-like activities [48]. Taken together, there is no doubt that rosiglitazone can enter into central nervous system.

## 5. Conclusions

We found that klotho expression in the medulla oblongata was reduced in STZ-diabetic rats. This is associated with the lower baroreflex response in STZ-diabetic rats because baroreflex was restored by the oral administration of rosiglitazone or treatment with the recombinant klotho through ICV infusion to higher cerebral klotho. Thus, rosiglitazone or TZDs is useful to reverse the reduced BRS through higher cerebral klotho in diabetic disorders.

## Figures and Tables

**Table 1:** Baseline level of the blood glucose, mean arterial pressure (MAP) and heart rate (HR), bradycardic and tachycardic peak, and HR range in STZ rats receiving rat IgG (IgG) or recombinant klotho (rKl).

Variable	Wistar	STZ	STZ + IgG	STZ + rKl
Blood glucose (mg/dL)	103 ± 4.3	363 ± 13.6***	362 ± 9.1***	368 ± 5.4***
MAP (mmHg)	112.8 ± 3.17	89.2 ± 2.74**	91.4 ± 3.82**	104.9 ± 7.21^#^
HR (bpm)	343 ± 7.2	281 ± 5.4***	283 ± 8.2***	308 ± 9.4^#^
Bradycardic peak (bpm)	243 ± 4.3	247 ± 7.2	243 ± 5.8	248 ± 4.7
Tachycardic peak (bpm)	442 ± 7.6	352 ± 7.4**	347 ± 6.6**	426 ± 5.4^##^
HR range (bpm)	194 ± 6.8	103 ± 8.7***	102 ± 5.3***	173 ± 8.9^##^

Values (mean ± SE) were obtained from each group of eight rats. *P < 0.05, **P < 0.01, and ***P < 0.001 compared to Wistar. ^#^ *P* < 0.05 and ^##^ *P* < 0.01 compared to STZ.

**Table 2:** Baseline level of the blood glucose, mean arterial pressure (MAP) and heart rate (HR), bradycardic and tachycardic peak, and HR range in STZ rats receiving the rosiglitazone (TZD) or not.

Variable	Wistar	STZ	STZ + TZD
Blood glucose (mg/dL)	98 ± 3.7	367 ± 11.4***	361 ± 10.3***
MAP (mmHg)	109.8 ± 4.4	87.6 ± 5.7**	95.4 ± 7.3^∗#^
HR (bpm)	351 ± 8.3	287 ± 4.8***	316 ± 7.4^∗#^
Bradycardic peak (bpm)	248 ± 7.3	242 ± 6.2	245 ± 4.3
Tachycardic peak (bpm)	437 ± 8.5	363 ± 4.7**	392 ± 11.6^∗#^
HR range (bpm)	188 ± 8.2	123 ± 4.9**	142 ± 7.3^∗#^

Values (mean ± SE) were obtained from each group of eight rats. *P < 0.05, **P < 0.01, and ***P < 0.001 compared to Wistar. ^#^ *P* < 0.05 compared to STZ.
